# Study on Magnetic and Plasmonic Properties of Fe_3_O_4_-PEI-Au and Fe_3_O_4_-PEI-Ag Nanoparticles

**DOI:** 10.3390/ma17020509

**Published:** 2024-01-21

**Authors:** Shuya Ning, Shuo Wang, Zhihui Liu, Naming Zhang, Bin Yang, Fanghui Zhang

**Affiliations:** 1 School of Electronic Information and Artificial Intelligence, Shaanxi University of Science and Technology, Xi’an 710021, China; ningshuya@sust.edu.cn (S.N.); wangshuo.99@sust.edu.cn (S.W.); lewis_zhihui@sust.edu.cn (Z.L.); zhangfanghui@sust.edu.cn (F.Z.); 2 Key Laboratory of Photonics Technology for Information, School of Electronic and Information Engineering, Xi’an Jiaotong University, Xi’an 710049, China; 3 State Key Laboratory of Electrical Insulation and Power Equipment, School of Electrical Engineering, Xi’an Jiaotong University, Xi’an 710049, China; yangbin.2008@stu.xjtu.edu.cn

**Keywords:** Fe_3_O_4_-PEI-M (M = Au or Ag) nanoparticles, plasmonic, magnetic

## Abstract

Magnetic–plasmonic nanoparticles (NPs) have attracted great interest in many fields because they can exhibit more physical and chemical properties than individual magnetic or plasmonic NPs. In this work, we synthesized Au- or Ag-decorated Fe_3_O_4_ nanoparticles coated with PEI (Fe_3_O_4_-PEI-M (M = Au or Ag) NPs) using a simple method. The influences of the plasmonic metal NPs’ (Au or Ag) coating density on the magnetic and plasmonic properties of the Fe_3_O_4_-PEI-M (M = Au or Ag) NPs were investigated, and the density of the plasmonic metal NPs coated on the Fe_3_O_4_ NPs surfaces could be adjusted by controlling the polyethyleneimine (PEI) concentration. It showed that the Fe_3_O_4_-PEI-M (M = Au or Ag) NPs exhibited both magnetic and plasmonic properties. When the PEI concentration increased from 5 to 35 mg/mL, the coating density of the Au or Ag NPs on the Fe_3_O_4_ NPs surfaces increased, the corresponding magnetic intensity became weaker, and the plasmonic intensity was stronger. At the same time, the plasmonic resonance peak of the Fe_3_O_4_-PEI-M (M = Au or Ag) NPs was red shifted. Therefore, there was an optimal coverage of the plasmonic metal NPs on the Fe_3_O_4_ NPs surfaces to balance the magnetic and plasmonic properties when the PEI concentration was between 15 and 25 mg/mL. This result can guide the application of the Fe_3_O_4_-M (M = Au or Ag) NPs in the biomedical field.

## 1. Introduction

At present, magnetic nanoparticles (NPs) have attracted great interest in many fields, such as bearing drugs [1,2], radionuclides [3,4], hyperthermia [5,6,7], active magnetic field targeting [8,9,10], protein purification [11,12,13], biosensors [14,15], and catalysis [16,17,18]. Researchers also further modify the surface of magnetic NPs with antibodies or proteins to expand their applications [19,20,21]. As such, Sohn et al. combined iron oxide nanoparticles with glucose transporter 1 antibody for vascular therapy [19]. Gawali et al. prepared magnetic nanoparticles conjugated with bovine serum albumin protein for magnetothermal therapy [21]. Unfortunately, magnetic NPs can easily aggregate, and it is difficult for them to couple with biomolecules due to the lack of functional groups, which limits the applications of magnetic nanomaterials [22]. In order to overcome these shortcomings, metal or metal oxide shells were coated on the surface of the magnetic nanomaterials to decorate the nanoparticles [23]. Noble metals (Au, Ag) are considered to be the most ideal coating material due to their unique optical properties, localized surface plasmon resonance (LSPR), high stability, biocompatibility, and easy surface functionalization [24,25,26,27]. Magnetic–plasmonic NPs, consisting of plasmonic metal materials (Au or Ag) coated onto the surfaces of magnetic NPs, can exhibit more physical and chemical properties than individual magnetic or plasmonic NPs, such as magnetic, plasmonic, biological, compatibility, chemical stability, and physicochemical properties [28,29,30,31]. They are considered as effective candidate materials for catalysts [32], sensors [33], antibacterial materials [34], cancer detection [35], and medical applications [36]. Therefore, it is of great significance to combine magnetic NPs with noble metal materials to generate nanoparticles that simultaneously exhibit both excellent magnetic and plasmonic characteristics. This has been the focus of many researchers in recent years. In 2020, Kou et al. prepared flower-shaped Fe_3_O_4_-Au NPs and found that the magnetic properties gradually decreased by increasing the amount of Au seeds [37]. Aarthi et al. synthesized Fe_3_O_4_/Ag NPs and studied the surface-enhanced Raman scattering (SERS), and the photocatalytic and antibacterial activities of the nanoparticles [38]. Oguzlar studied Fe_3_O_4_ and Fe_3_O_4_@Ag NPs to improve the oxygen sensitivity of ruthenium dyes [39]. In 2021, Du et al. synthesized Fe_3_O_4_@Au core-shell NPs and studied both the magnetic and optical properties derived from the Fe_3_O_4_ NPs and the Au nano-shells [40]. Salimi et al. prepared Au-Fe_3_O_4_ NPs and studied the nano-morphology and formation process of the Au-Fe_3_O_4_ NPs [41]. In 2022, Lv et al. prepared the Fe_3_O_4_@Au NPs, which showed LSPR absorption in the near-infrared region [42]. Mikoliunaite et al. prepared Fe_3_O_4_@Ag NPs and found that their plasmonic resonance could be varied from 470 to 800 nm by changing the volume of the Ag colloid solution [43]. In 2023, Ravichandran et al. synthesized Fe_3_O_4_/Ag NPs and studied the dye removal rate of the Fe_3_O_4_/Ag NPs in the presence of a reducing agent [44]. According to the above works, most of the research on Fe_3_O_4_-M (M = Au or Ag) NPs was limited to a single particle type or physical property, and studies on the relationships between the coverage of the plasmonic metal NPs on the magnetic NPs surfaces and both the magnetic and plasmonic properties are lacking. Therefore, it is important to systematically investigate the magnetic and plasmonic characteristics of Fe_3_O_4_-M (M = Au or Ag) NPs.

**Scheme 1:**
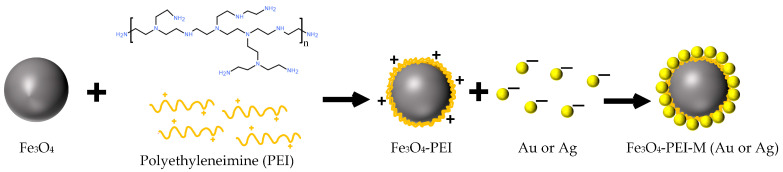
Schematic of the preparation of Fe_3_O_4_-PEI-M (M = Au or Ag) nanoparticles.

In this work, we prepared the Au- or Ag-decorated Fe_3_O_4_ nanoparticles coated with PEI (Fe_3_O_4_-PEI-M (M = Au or Ag) NPs) using a simple method. Fe_3_O_4_ NPs were synthesized through a co-precipitation method, and plasmonic metal (Au or Ag) NPs were prepared using a chemical reduction method. Fe_3_O_4_ NPs and plasmonic metal NPs were mixed at room temperature to form Fe_3_O_4_-PEI-M (M = Au or Ag) NPs. In this process, positively charged PEI was assembled onto negatively charged Fe_3_O_4_ through electrostatic self-assembly. Then, negatively charged plasmonic metal NPs were electrostatically bound to the positively charged PEI-coated Fe_3_O_4_ NPs, resulting in a formation of Fe_3_O_4_-PEI-M (M = Au or Ag) NPs (Scheme 1). The density of the plasmonic metal NPs coated on the Fe_3_O_4_ NPs was adjusted by controlling the concentration of polyethyleneimine (PEI), and the effects of the plasmonic metal NPs’ density on the magnetic and plasmonic properties of the Fe_3_O_4_-PEI-M (M=Au or Ag) NPs were studied. The relationships between the coating density of the plasmonic metal NPs and both the magnetic and plasmonic properties were established. This work can provide guidance for the application of Fe_3_O_4_-M (M = Au or Ag) NPs in the biomedical field.

## 2. Materials and Methods

### 2.1. Materials

Ferric chloride hexahydrate (FeCl_3_·6H_2_O), ferrous chloride tetrahydrate (FeCl_2_·4H_2_O), and ammonium hydroxide (28–30% NH_3_) were obtained from Merck (Shanghai, China). Polyethyleneimine (PEI, branched, M_w_ ≈ 10,000 g/mol), sodium hydroxide (NaOH), chloroauric acid (HAuCl_4_), silver nitrate (AgNO_3_), sodium borohydride (NaBH_4_), and sodium citrate (C_6_H_5_Na_3_O_7_·2H_2_O) were obtained from Aladdin (Shanghai, China). All chemicals were used as received with no further purification.

### 2.2. Synthesis of Fe_3_O_4_ Nanoparticles

Fe_3_O_4_ nanoparticles were synthesized through a co-precipitation method [45]. A total of 4.25 g of FeCl_3_·6H_2_O and 2 g of FeCl_3_·6H_2_O were added to 200 mL of ultrapure water. In an oxygen-free environment, the mixture was heated to 70 °C with stirring at 800 rpm and maintained at this temperature for 1 h. A total of 10 mL of ammonia solution (25% wt) was added into the mixture. A black precipitate was produced, indicating the formation of Fe_3_O_4_ NPs. The resulting solution was stirred for another 15 min. The nanoparticles were purified by means of magnetic separation five times and then redispersed in 200 mL of ultrapure water for further use.

### 2.3. Synthesis of Au Nanoparticles

Au nanoparticles were synthesized through NaBH_4_ reduction of chloroauric acid [29]. Firstly, 0.5 mL of 0.01 M HAuCl_4_ was mixed with 0.5 mL of a 0.01 M trisodium citrate solution. Then, the mixture was added into 18 mL of ultrapure water and stirred at 1200 rpm. A total of 0.5 mL of an ice 0.04 M NaBH_4_ solution was quickly added with vigorous stirring at room temperature. When the solution turned pink, indicating the formation of Au NPs, the solution was allowed to stand for 2 h. The particles were collected by centrifugation at 14,000 rpm for 10 min. The Au nanoparticles were stored in the fridge until further use.

### 2.4. Synthesis of Ag Nanoparticles

Ag nanoparticles were prepared through the NaBH_4_ reduction of silver nitrate [46]. Briefly, 10 mL of a 1 mM AgNO_3_ solution was mixed with 0.8 mL of 40 mM sodium citrate solution used as stabilizer. A total of 0.2 mL of an ice 0.06 M NaBH_4_ solution was added into the mixture, and the mixture was stirred at 1200 rpm for 15 min. After the solution turned dark yellow, indicating the formation of Ag NPs, the Ag nanoparticles were collected by means of centrifugation at 14,000 rpm for 10 min. The Ag nanoparticles were stored in the fridge until further use.

### 2.5. Synthesis of Fe_3_O_4_-PEI-M (M = Au or Ag) NPs

Firstly, 2 mL of the Fe_3_O_4_ NPs suspension (1 mg/mL) was mixed with 20 mL of the PEI solution. After 60 min of ultrasonic treatment, the mixture was left to stand for half an hour [47]. Subsequently, magnetic separation was employed for washing the nanoparticles. The PEI-coated Fe_3_O_4_ NPs were finally redispersed in 2 mL of ultrapure water. Then, the 0.5 mL PEI-coated Fe_3_O_4_ NPs solution was mixed with 4.5 mL of the plasmonic metal (Au or Ag) NPs solution. The mixture was continuously stirred at 2000 rpm for 45 min. Finally, the Au- or Ag-decorated Fe_3_O_4_ nanoparticles coated with PEI were collected by magnetic separation and washed with ultrapure water three times. By varying the concentrations of the PEI solution, the adhesion density of the plasmonic metal NPs on the Fe_3_O_4_ NPs surfaces could be changed.

### 2.6. Electric Field Simulation

To investigate the plasmonic properties of nanoparticles, the electric field distributions of the nanoparticles were simulated using the finite element method with the commercial software COMSOL 5.5. The excitation beam was a plane wave polarized along the x-axis with the plasmonic resonance wavelength of the nanoparticle, and the permittivities of Au and Ag were taken from the experimental data of Johnson and Christy [48]. The mesh size was set to 2 nm.

### 2.7. Characterization

The morphology and structure of the nanoparticles were observed by scanning electron microscopy (SEM, JSM-7000F, JEOL, Tokyo, Japan) and transmission electron microscopy (TEM, JEM-2100, JEOL, Tokyo, Japan). Hydrodynamic diameter and zeta-potential measurements were carried out using dynamic light scattering (DLS, Delsa, Palmdale, CA, USA). The crystalline structure of the nanoparticles was analyzed by X-ray powder diffraction (XRD, D8 ADVANCE, Bruker, Bremen, Germany). UV–visible absorption spectra were obtained using a UV–vis spectrophotometer (Cary 5000, Agilent, Santa Clara, CA, USA). Magnetization measurements of the nanoparticles were performed using a vibrating sample magnetometer (MPMS-squid VSM-094, Quantum Design, San Diego, CA, USA).

## 3. Results and Discussion

### 3.1. Study on the Plasmonic Metal Nanoparticles

#### 3.1.1. Au Nanoparticles

**Figure 1:**
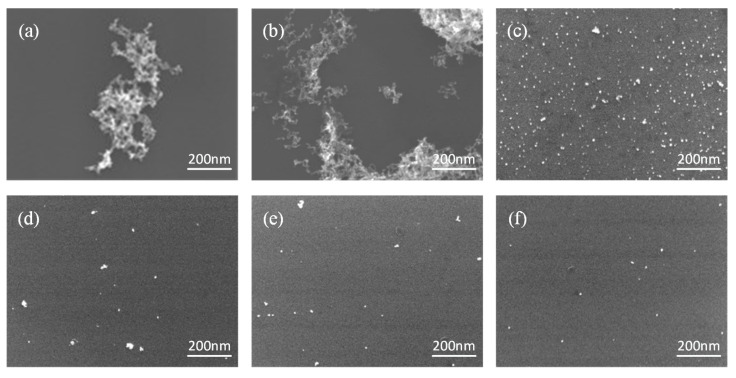
SEM images of the Au NPs prepared with (**a**) 4 M, (**b**) 2 M, (**c**) 1 M, (**d**) 0.4 M, (**e**) 0.08 M, and (**f**) 0.04 M NaBH_4_ solution.

To prepare suitable Au nanoparticles for coating Fe_3_O_4_ NPs’ surfaces, we investigated the influence of the reducing agent (NaBH_4_) concentrations on the Au nanoparticles. Figure 1 shows the SEM images of the Au NPs synthesized with NaBH_4_ concentrations ranging from 4 to 0.04 M. We found that the synthesized Au nanoparticles formed agglomerates with an average diameter of about 12 nm when the NaBH_4_ concentration was 4 M (Figure 1a). With a decrease in the NaBH_4_ concentration from 4 to 0.04 M, the Au NPs became more diffuse, and the particle size became more uniform. When the NaBH_4_ concentration was 0.04 M, the average diameter of the Au NPs was about 10 nm, as shown in Figure 1f, and there was excellent dispersion, which was beneficial for attachment on the Fe_3_O_4_ NPs’ surfaces. Figure 1 shows that the particles size increased and more easily agglomerated with the increase in NaBH_4_ concentration. This is attributed to the over-reduction of HAuCl_4_ in the case of excessive amounts of reducing agent [49,50].

#### 3.1.2. Ag Nanoparticles

**Figure 2:**
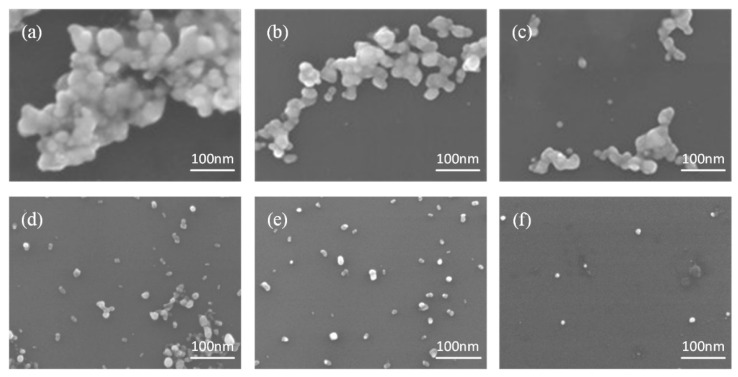
SEM images of the Ag NPs prepared with (**a**) 8.5 M, (**b**) 0.85 M, (**c**) 0.43 M, (**d**) 0.17 M, (**e**) 0.08 M, and (**f**) 0.06 M NaBH_4_ solution.

In addition, we also investigated the Ag NPs, which were prepared using trisodium citrate as a stabilizer and NaBH_4_ to reduce AgNO_3_. Figure 2 shows the SEM images of the Ag NPs prepared with different NaBH_4_ concentrations. We found that the synthesized Ag nanoparticles formed agglomerates with an average diameter of about 25 nm when the NaBH_4_ concentration was 8.5 M (Figure 2a). With a decrease in the NaBH_4_ concentration from 8.5 to 0.06 M, the Ag NPs became more diffuse, and the size became more uniform [51]. This was due to more Ag atoms being produced with the increase in NaBH_4_ concentration [49]. When the concentration of NaBH_4_ was 0.06 M, the average diameter of the Au NPs was about 10 nm, as shown in Figure 2f, and there was excellent dispersion, which is desirable for the further preparation of Ag-decorated Fe_3_O_4_ NPs.

### 3.2. Study on the Properties of the Fe_3_O_4_-PEI-Au Nanoparticles

#### 3.2.1. Fe_3_O_4_-PEI-Au Nanoparticles

**Figure 3:**
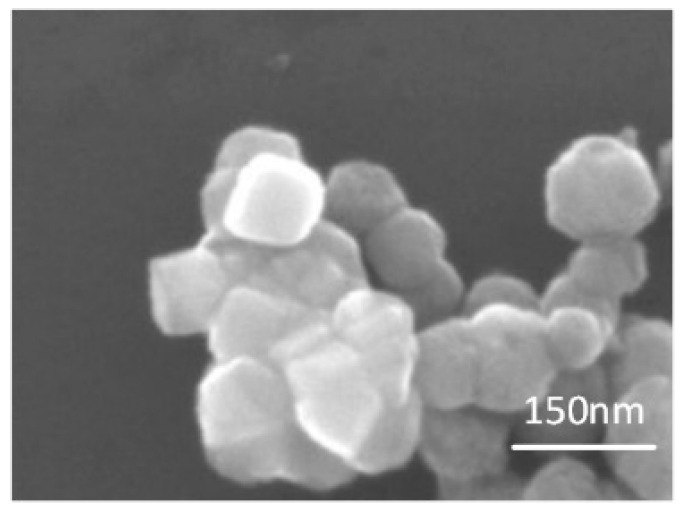
SEM image of the Fe_3_O_4_ NPs.

In order to explore the magnetic and plasmonic properties of the Au-decorated Fe_3_O_4_ nanoparticles, we synthesized the Fe_3_O_4_-PEI-Au NPs with different densities of Au NPs on the Fe_3_O_4_ NPs surfaces. Figure 3 shows the SEM image of the Fe_3_O_4_ NPs, and the average size was about 80 nm in diameter.

**Figure 4:**
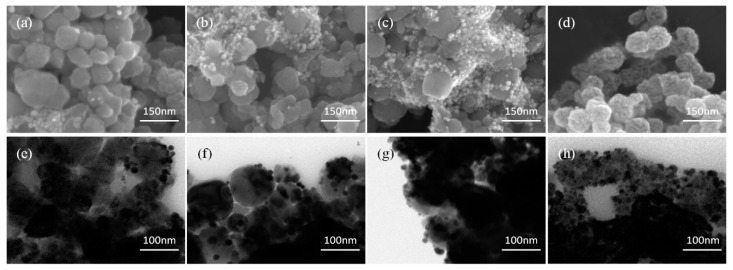
SEM images of the Fe_3_O_4_-PEI-Au NPs treated with (**a**) 5, (**b**) 15, (**c**) 25, and (**d**) 35 mg/mL PEI solution. The corresponding TEM images of the Fe_3_O_4_-PEI-Au NPs treated with (**e**) 5, (**f**) 15, (**g**) 25, and (**h**) 35 mg/mL PEI solution.

**Figure 5:**
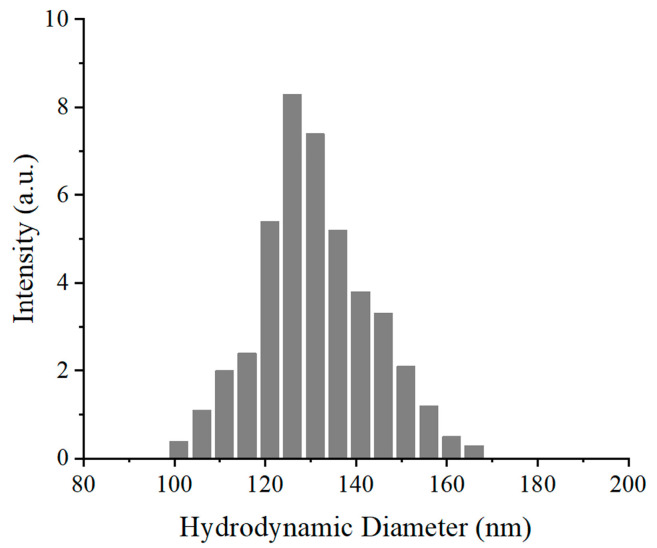
Hydrodynamic diameter distribution of Fe_3_O_4_-PEI-Au NPs (PEI: 35 mg/mL).

The coating density of Au NPs on the Fe_3_O_4_ NPs surfaces was controlled by changing the PEI concentration. PEI is used as a bonding material. It is a water-soluble cationic polymer which contains amino and imino groups in each polymer chain. When the polymer PEI is dispersed in aqueous solution, each polymer chain is positively charged due to the amino and imino groups [52]. When the Fe_3_O_4_ NPs are immersed in a PEI solution, positively charged PEI is assembled onto the negatively charged Fe_3_O_4_ through electrostatic self-assembly, leading to the formation of a stable polyelectrolyte layer. Subsequently, the negatively charged Au NPs are easily electrostatically bound to the positively charged PEI-coated Fe_3_O_4_ NPs, resulting in a formation of the Fe_3_O_4_-PEI-Au NPs [53]. Figure 4 shows the SEM and TEM images of the Au-decorated Fe_3_O_4_ NPs treated with different concentrations of PEI solutions. It shows that 10 nm diameter Au NPs were attached to the surfaces of 80 nm diameter Fe_3_O_4_ NPs. DLS measurements depicted that the hydrodynamic diameter of the Fe_3_O_4_-PEI-Au NPs is about 131 nm (shown in Figure 5), and the zeta potential is around +18.4 mV. When the Fe_3_O_4_ NPs were treated with a 5 mg/mL PEI solution, a small amount of Au NP was coated on the Fe_3_O_4_ NPs’ surfaces, as shown in Figure 4a,e. With an increase in the PEI concentration from 5 to 35 mg/mL, the coating density of the Au NPs gradually increased, and thus the coverage of the Au NPs on the Fe_3_O_4_ NPs surfaces increased. This is attributed to a greater amount of NH_2_ groups provided by PEI that can adsorb more Au NPs with an increase in the PEI concentration.

#### 3.2.2. X-ray Diffraction (XRD) Analysis

**Figure 6:**
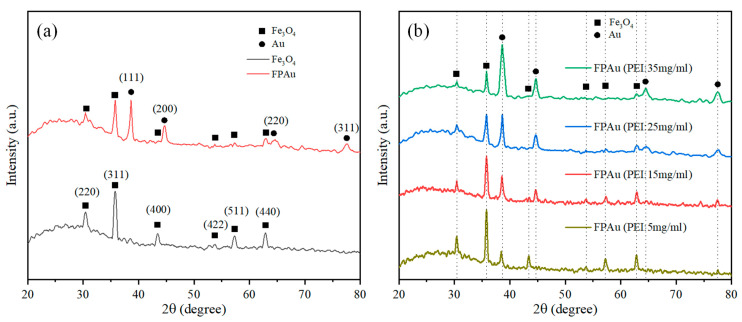
XRD spectra of the (**a**) Fe_3_O_4_ and Fe_3_O_4_-PEI-Au (FPAu) NPs, and (**b**) Fe_3_O_4_-PEI-Au (FPAu) NPs treated with 5~35 mg/mL PEI solution.

An X-ray diffractometer was used to characterize the crystal structures of the Fe_3_O_4_ and Fe_3_O_4_-PEI-Au NPs. As shown in Figure 6a, the XRD spectrum of Fe_3_O_4_ showed six diffraction peaks at 2θ values of 30.43°, 35.78°, 43.44°, 53.79°, 57.34°, and 62.89°, corresponding to the diffraction peaks located at (220), (311), (400), (422), (511), and (440). All these diffraction peaks correspond to the face-centered cubic structure of Fe_3_O_4_. Compared to that of Fe_3_O_4_, the XRD spectrum of the Fe_3_O_4_-PEI-Au NPs exhibited additional peaks at 38.62°, 44.67°, 64.61°, and 77.62°, which match well with the (111), (200), (220), and (311) faces of Au. This result is consistent with that reported in the literature [54]. Therefore, the X-ray diffraction spectra further confirmed that Au NPs were successfully loaded onto the Fe_3_O_4_ NPs surfaces. In addition, in Figure 6b, it is implied that with an increase in the PEI concentration, the characteristic peak intensities of the Au NPs gradually increased, while the characteristic peak intensities of the Fe_3_O_4_ NPs gradually decreased. This is because of the increase in the density of Au NPs coated on the Fe_3_O_4_ NPs surfaces. No peaks of other impurities were detected in the XRD spectra, indicating a high purity of the materials.

#### 3.2.3. Analysis of Magnetic and Plasmonic Properties

**Figure 7:**
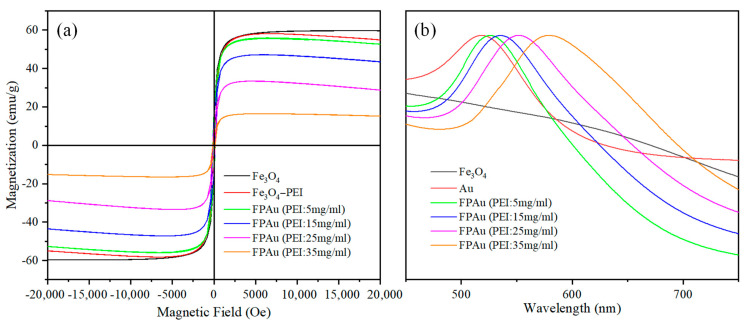
(**a**) Hysteresis loops and (**b**) normalized absorption spectra of the Fe_3_O_4_ and Fe_3_O_4_−PEI−Au (FPAu) NPs treated with 5~35 mg/mL PEI solution.

Firstly, the magnetic properties of the Fe_3_O_4_-PEI-Au NPs with different coverages of the Au NPs on the Fe_3_O_4_ NPs surfaces were studied. Figure 7a exhibits the hysteresis loops of the Fe_3_O_4_-PEI-Au NPs with different Au NP densities, controlled by using different PEI concentrations (the magnetic field scanning range was 20,000 g at 300 K). All the curves for the Fe_3_O_4_ and Fe_3_O_4_-PEI-Au NPs have similar shapes. The saturation magnetization of the Fe_3_O_4_ NPs was 60 emu/g, which is same as that of Fe_3_O_4_ NPs in a previous study [55]. After treatment with the PEI solution, the saturation magnetization of the Fe_3_O_4_-PEI NPs decreased to 57 emu/g. This value is a little smaller than the one previously reported for PEI-coated Fe_3_O_4_ NPs [56]. This may be due to the agglomeration of particles and the interaction between them [55]. Furthermore, for the Fe_3_O_4_-PEI-Au NPs, when the PEI concentration increased from 5 to 35 mg/mL, the Au NP coating density increased, and the saturation magnetization of the Fe_3_O_4_-PEI-Au NPs decreased from 55 to 16 emu/g, as shown in Table 1. The reduction in saturation magnetization of the Fe_3_O_4_ NPs due to the introduction of plasmonic metal NPs could be demonstrated in reference [54]. Therefore, with the increase in the density of the Au NPs on the Fe_3_O_4_ NPs surfaces, the shielding effect of the Au NPs on the Fe_3_O_4_ NPs increases.

**Figure 8:**
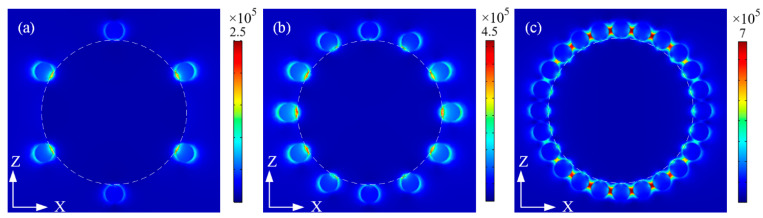
Electric field distributions of the Fe_3_O_4_ NP coating with Au NPs of (**a**) sparse, (**b**) medium, and (**c**) dense.

Due to Fe_3_O_4_-PEI-Au NPs with different Au NP densities exhibiting different magnetic and plasmonic properties, we also investigated the plasmonic properties of the Fe_3_O_4_-PEI-Au NPs. Figure 7b shows the absorption spectra of the Fe_3_O_4_-PEI-Au NPs with different Au NP densities. The Fe_3_O_4_ NPs had no absorption peak in the visible region, which is consistent with the result from a previous study [57]. The absorption peak of the Au NPs was located at 519 nm. When the PEI concentration increased from 5 to 35 mg/mL, the Au NP coating density increased and the absorption peak of the Fe_3_O_4_-PEI-Au NPs red shifted from 526 to 578 nm (shown in Table 1) because of the decrease in the gap distance between the Au NPs. To further investigate the influence of the Au NP coating density on the enhanced electric field of the nanoparticles, three-dimensional models of the Fe_3_O_4_ nanoparticle decorated with different Au NP densities were built. Many researchers calculated electric field characteristics by quantum method [58], or classical method [59,60]. Here, the electric field distributions were simulated using the finite element method with the commercial software COMSOL 5.5. Figure 8 shows the electric field distributions of Au-decorated Fe_3_O_4_ NPs with Au NP coating densities ranging from sparse to dense. It was observed that with an increase in the Au NP coating density, the electric field became stronger, and the plasmonic coupling between the adjacent nanoparticles became stronger, indicating that the plasmonic properties were enhanced.

### 3.3. Study on the Properties of the Fe_3_O_4_-PEI-Ag Nanoparticles

#### 3.3.1. Fe_3_O_4_-PEI-Ag Nanoparticles

**Figure 9:**
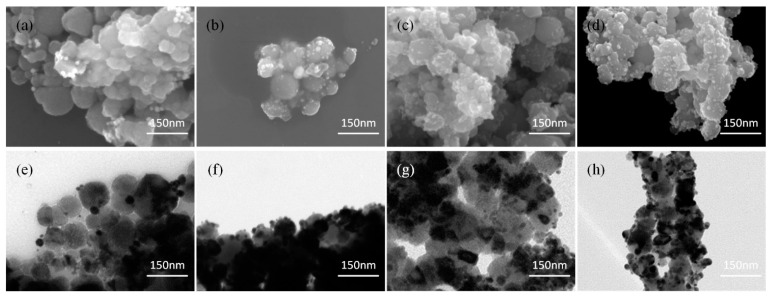
SEM images of the Fe_3_O_4_-PEI-Ag NPs treated with (**a**) 5, (**b**) 15, (**c**) 25, and (**d**) 35 mg/mL PEI solution. The corresponding TEM images of the Fe_3_O_4_-PEI-Ag NPs treated with (**e**) 5, (**f**) 15, (**g**) 25, and (**h**) 35 mg/mL PEI solution.

**Figure 10:**
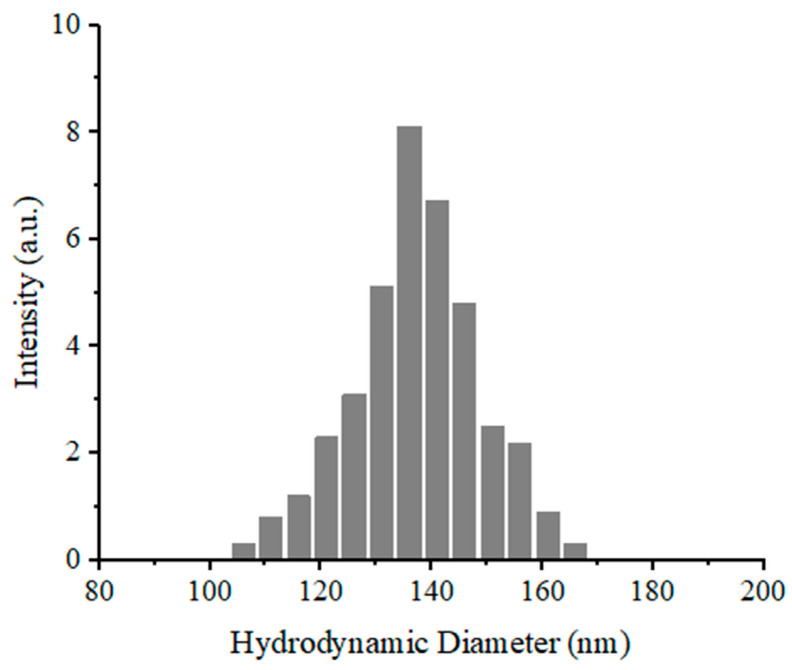
Hydrodynamic diameter distribution of Fe_3_O_4_-PEI-Ag NPs (PEI: 35 mg/mL).

According to Figure 7b, the plasmonic resonance peak of the Fe_3_O_4_-PEI-Au NPs is located at about 526 nm. However, when a shorter wavelength of plasmonic resonance is needed, Fe_3_O_4_-PEI-Ag NPs are more suitable; thus, we also need to study their magnetic and plasmonic properties. Here, we synthesized Fe_3_O_4_-PEI-Ag NPs using the same methods that were utilized to prepare the Fe_3_O_4_-PEI-Au NPs. Similarly, positively charged PEI binds to negatively charged Fe_3_O_4_ by electrostatic self-assembly, and negatively charged Ag nanoparticles combine with positively charged PEI-coated Fe_3_O_4_ nanoparticles to form Fe_3_O_4_-PEI-Ag NPs. Figure 9 shows SEM and TEM images of the Ag-decorated Fe_3_O_4_ NPs treated with PEI concentrations ranging from 5 to 35 mg/mL. It was observed that 10 nm diameter Ag NPs were attached to the surface of 80 nm diameter Fe_3_O_4_ NPs. DLS measurements show that the hydrodynamic diameter of the Fe_3_O_4_-PEI-Ag NPs is about 137 nm (as shown in Figure 10) and the zeta potential is around +19.1 mV. When the Fe_3_O_4_ NPs were treated with a 5 mg/mL PEI solution, a small amount of Ag NP was coated on the Fe_3_O_4_ NP surfaces, as shown in Figure 9a,e. When the concentration of PEI increased from 5 to 35 mg/mL, the coverage of the Ag NPs on the Fe_3_O_4_ NPs surfaces increased gradually. This is because of the greater amount of NH_2_ groups provided by the PEI leading to an increased number of Ag NPs attached on the Fe_3_O_4_ surface with the increase in PEI concentration.

#### 3.3.2. X-ray Diffraction Analysis

**Figure 11:**
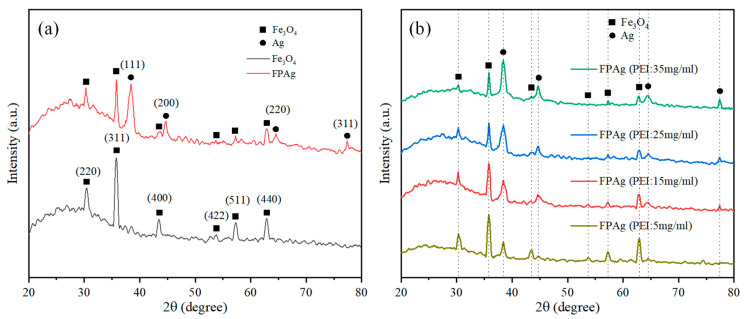
XRD spectra of the (**a**) Fe_3_O_4_ and Fe_3_O_4_-PEI-Ag (FPAg) NPs, and (**b**) Fe_3_O_4_-PEI-Ag (FPAg) NPs treated with 5~35 mg/mL PEI solution.

The crystalline structures of the Fe_3_O_4_ and Fe_3_O_4_-PEI-Ag NPs were characterized using an X-ray diffractometer. As shown in Figure 11a, the positions and relative intensities of all diffraction peaks are in good agreement with the standard Fe_3_O_4_ and Ag NP diffraction peaks. Compared to the Fe_3_O_4_ NPs, there were four more main peaks in the Fe_3_O_4_-PEI-Ag NP spectrum, which can be clearly observed at 2θ values of 38.42°, 44.63°, 64.53°, and 77.38°, corresponding to the reflection of the (111), (200), (220), and (311) crystal planes of Ag [61]. Therefore, the X-ray diffraction spectra further confirmed that Ag NPs were successfully loaded on the Fe_3_O_4_ NPs surfaces. In addition, Figure 11b shows that, with an increase in the PEI concentration, the characteristic peak intensities of the Ag NPs gradually increased, while that of the Fe_3_O_4_ NPs gradually decreased. This is because of the increase in the density of Ag NPs coated on the Fe_3_O_4_ NPs surfaces. No peaks of other impurities were detected in the XRD spectra, indicating high purity of the materials.

#### 3.3.3. Analysis of Magnetic and Plasmonic Properties

**Figure 12:**
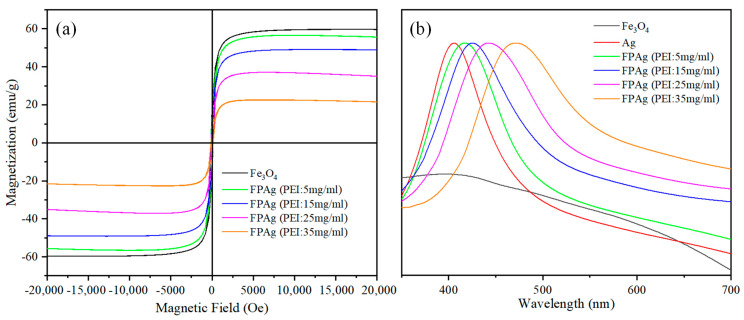
(**a**) Hysteresis loops and (**b**) normalized absorption spectra of the Fe_3_O_4_ and Fe_3_O_4_−PEI−Ag (FPAg) NPs treated with 5~35 mg/mL PEI solution.

Figure 12a illustrates the hysteresis loops of the Ag-decorated Fe_3_O_4_ NPs treated with different PEI concentrations. It can be observed that the curves of the Fe_3_O_4_ and Fe_3_O_4_-PEI-Ag NPs exhibit similar trends. The saturation magnetization of the Fe_3_O_4_ NPs was 60 emu/g. When the PEI concentration increased from 5 to 35 mg/mL, the Ag NP coating density increased, and the saturation magnetization of the Fe_3_O_4_-PEI-Ag NPs decreased from 56 to 22 emu/g, as shown in Table 2. Therefore, with an increase in the Ag NP density, the shielding effect of the Ag NPs coated on the Fe_3_O_4_ NPs increases.

**Figure 13:**
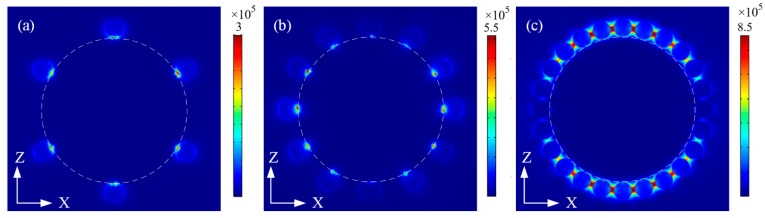
Electric field distributions of the Fe_3_O_4_ NP coating with Ag NPs of (**a**) sparse, (**b**) medium, (**c**) dense.

Figure 12b shows the absorption spectra of the Ag and Fe_3_O_4_-PEI-Ag NPs. It shows that 10 nm Ag NPs had a plasmonic absorption peak of 406 nm. When the PEI concentration increased from 5 to 35 mg/mL, the absorption peak of the Fe_3_O_4_-PEI-Ag NPs red shifted from 417 to 472 nm with the increase in the Ag NP coating density (Table 2). This is due to the decrease in the gap distance between the Ag NPs. To further study the effect of Ag NP coating density on the enhanced electric field of the nanoparticles, the electric field distributions were simulated using the finite element method. Figure 13 illustrates the electric field distributions of Ag-decorated Fe_3_O_4_ NPs with varying Ag NP coating densities ranging from sparse to dense. It was found that, as the coating density increased, the electric field became stronger. Therefore, according to the analysis of the magnetic and plasmonic properties of Au- or Ag-decorated Fe_3_O_4_ NPs treated with different PEI concentrations, as shown in Figure 7, Figure 8, Figure 12, and Figure 13, it is implied that when the PEI concentration is low, such as 5 mg/mL, the coverage of plasmonic metal NPs on the surface of Fe_3_O_4_ NPs is minimal. This results in a weak shielding effect of plasmonic metal NPs, leading to a strong magnetic field intensity but a weak plasmonic intensity in Fe_3_O_4_-PEI-M (M = Au or Ag) NPs. Conversely, when the PEI concentration is high (such as 35 mg/mL), the coverage of the plasmonic metal NPs is too large, the shielding effect of the plasmonic metal NPs is strong, the magnetic intensity becomes weak, and the plasmonic intensity becomes strong. Therefore, when the PEI concentration is between 15 and 25 mg/mL, there is an optimal coverage of the plasmonic metal NPs on the Fe_3_O_4_ NP surfaces to balance the magnetic and plasmonic properties.

## 4. Conclusions

In conclusion, we synthesized Fe_3_O_4_-PEI-M (M = Au or Ag) NPs using a simple method. The density of the plasmonic metal NPs coated on the Fe_3_O_4_ NPs surfaces was adjusted by controlling the PEI concentration. We investigated the effects of the Au or Ag NP coating density on the magnetic and plasmonic properties of the Fe_3_O_4_-PEI-M (M = Au or Ag) NPs. We found that, for the Fe_3_O_4_-PEI-M (M = Au or Ag) NPs, with an increase in the plasmonic metal NP density, the saturation magnetization decreased, the plasmonic intensity increased, and the plasmonic absorption peak was red shifted. When the concentration of PEI was between 15 and 25 mg/mL, Fe_3_O_4_-PEI-M (M = Au or Ag) NPs exhibited both excellent magnetic and plasmonic properties. The relationships among the coating density of the plasmonic metal NPs and both magnetic and plasmonic properties were established. This work can provide guidance for the application of Fe_3_O_4_-M (M = Au or Ag) NPs in the biomedical field.

## Figures and Tables

**Table 1:** Absorption peak wavelength and saturation magnetization of the Fe_3_O_4_, Au, Fe_3_O_4_-PEI-Au nanoparticles treated with different PEI concentrations.

	Fe_3_O_4_	Au	Fe_3_O_4_-PEI-Au PEI: 5 mg/mL	Fe_3_O_4_-PEI-Au PEI: 15 mg/mL	Fe_3_O_4_-PEI-Au PEI: 25 mg/mL	Fe_3_O_4_-PEI-Au PEI: 35 mg/mL
Absorption peak (nm)	/	519	526	535	552	578
Saturation magnetization (emu/g)	60	/	55	46	31	16

**Table 2:** Absorption peak and saturation magnetization of Fe_3_O_4_, Ag, and Fe_3_O_4_-PEI-Ag nanoparticles treated with different PEI concentrations.

	Fe_3_O_4_	Ag	Fe_3_O_4_-PEI-Ag PEI: 5 mg/mL	Fe_3_O_4_-PEI-Ag PEI: 15 mg/mL	Fe_3_O_4_-PEI-Ag PEI: 25 mg/mL	Fe_3_O_4_-PEI-Ag PEI: 35 mg/mL
Absorption peak (nm)	/	406	417	425	442	472
Saturation magnetization (emu/g)	60	/	56	49	36	22

## Data Availability

Data are contained within the article.
